# Supplementary material for: Hub Genes in Non-Small Cell Lung Cancer Regulatory Networks
Source: Biomolecules. 2022 Nov 29;12(12):1782. doi: 10.3390/biom12121782 (PMC9776006; doi:10.3390/biom12121782)
Supplement: Supplementary file 1 [file biomolecules-12-01782-s001.zip › Supplementary file 5.pdf]

The seven selected genes (*BUB3, PGAM1, DNM1L, STRAP, EIF2S1, KPNB1, NMT1*) were ranked within the top 10th percentile in at least one of the following seven networks: CNV-CNV network (GSE31800), Gene co-expression network (GSE31800), CNV-mediated gene expression network (GSE28582), Gene co-expression network (GSE28582), Gene co-expression network in Xu's LUAD tumors, Gene co-expression network in Xu's LUAD NATs, and Protein co-expression network in Xu's LUAD tumors. The following Venn diagrams showed the relationships among the seven genes in each network. The numbers in the plots show the number of common genes that have associations with the seven genes; the blanks in the plots mean there are no genes there.

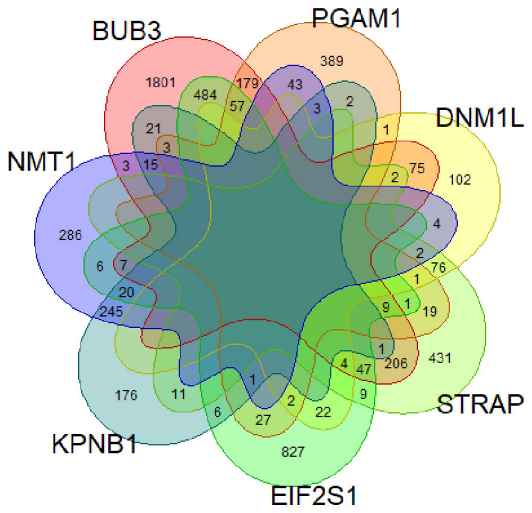

Figure S1. CNV-CNV network in GSE31800.

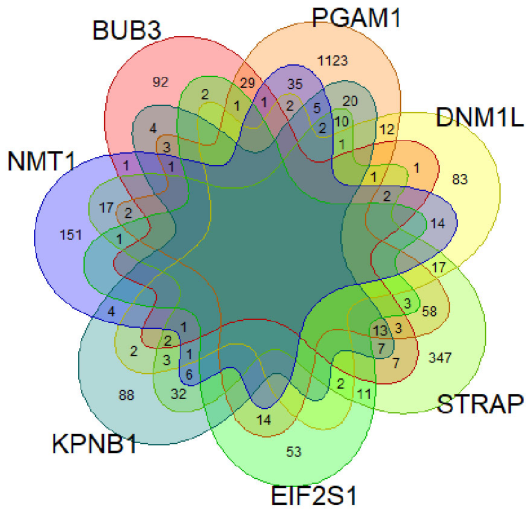

Figure S2. Gene co-expression network in GSE31800.

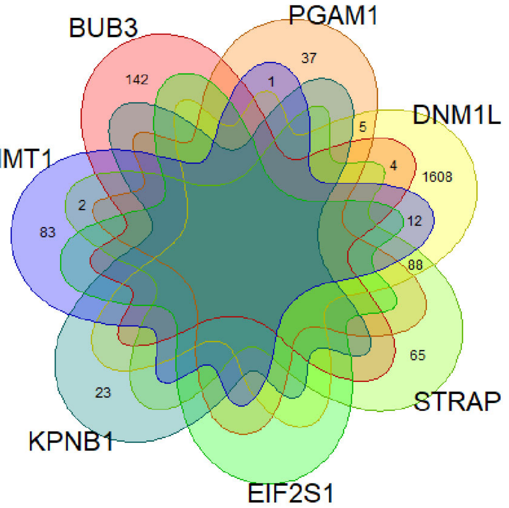

Figure S3. CNV-mediated gene expression network in GSE28582 – seven genes as regulatory genes.

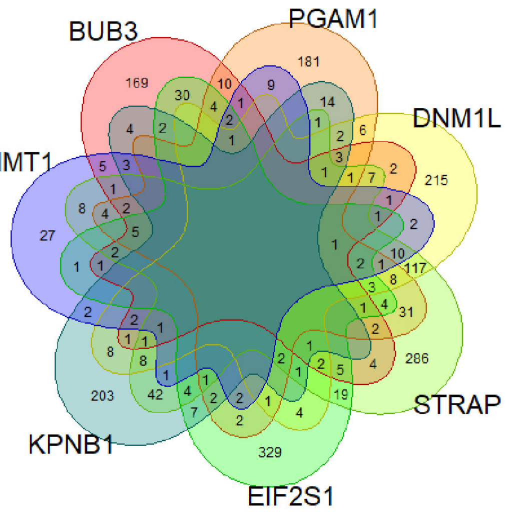

Figure S4. CNV-mediated gene expression network in GSE28582 – seven genes as regulated genes.

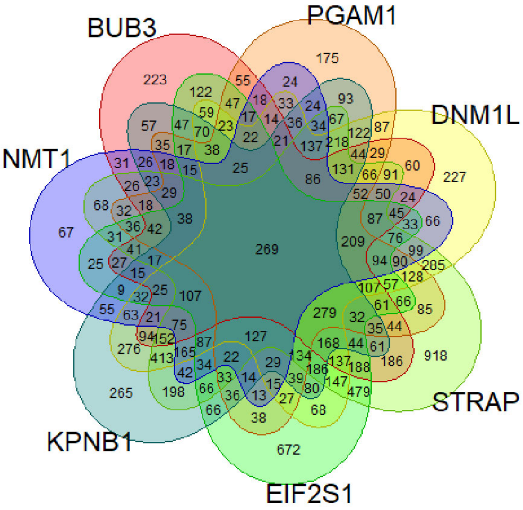

Figure S5. Gene co-expression network in GSE28582.

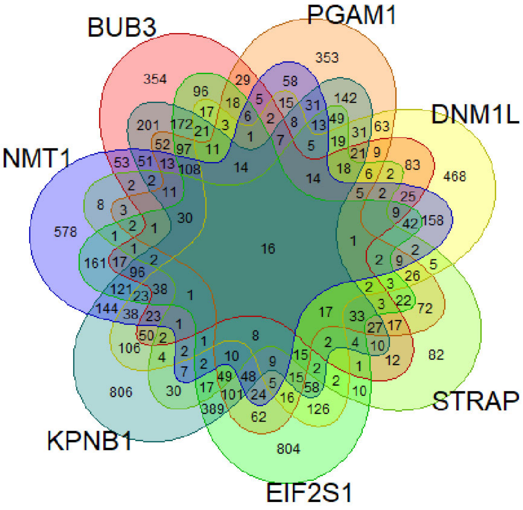

Figure S6. Gene co-expression network in Xu's LUAD tumors.

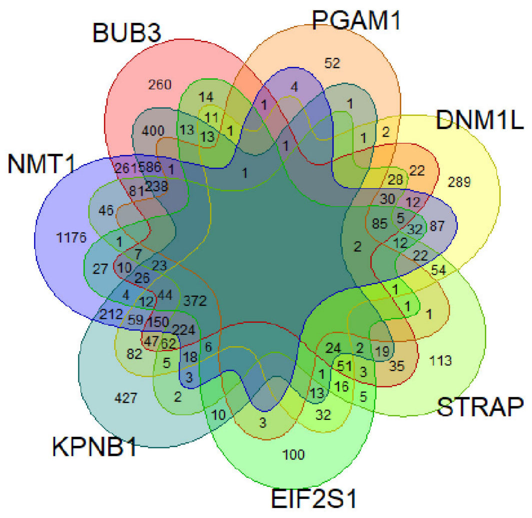

Figure S7. Gene co-expression network in Xu's LUAD NATs.

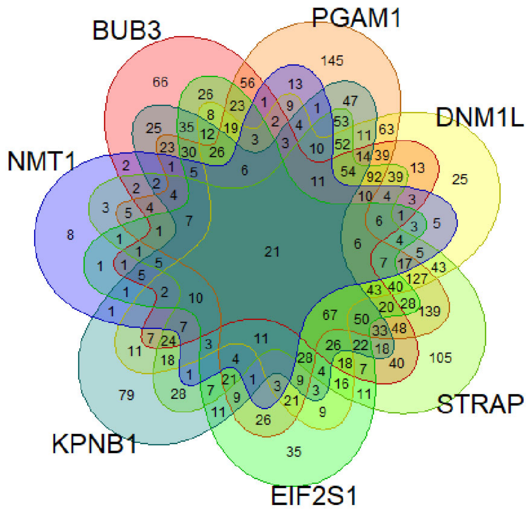

Figure S8. Protein co-expression network in Xu's LUAD tumors.
